# Comorbidities associated with non- healing of plantar ulcers in leprosy patients

**DOI:** 10.1371/journal.pntd.0008393

**Published:** 2020-06-29

**Authors:** Brahmaiah Upputuri, Aparna Srikantam, Raja Sriswan Mamidi

**Affiliations:** 1 Clinical Division, Blue Peter Public Health and Research Centre-LEPRA Society, Hyderabad, Telangana, India; 2 Clinical and Laboratory Research Division, Blue Peter Public Health and Research Centre-LEPRA Society, Hyderabad, Telangana, India; 3 Clinical Epidemiology, ICMR-National Institute of Nutrition, Hyderabad, Telangana, India; Hospital Infantil de Mexico Federico Gomez, MEXICO

## Abstract

**Background:**

Non-healing plantar ulcers are one of the significant causes of disability in leprosy patients. Plantar ulcers often take months or years to heal, affecting the patient’s quality of life. Presence of comorbid conditions in these patients can delay wound healing. The study aimed to evaluate the role of associated comorbid conditions as risk factors in ulcer healing.

**Methodology/Principal findings:**

A total of 66 leprosy patients with plantar ulcers registered at LEPRA Society-Blue Peter Public Health and Research Center (BPHRC), Hyderabad, India from June 2018 to June 2019 were studied. Comprehensive clinical assessment was done, including screening for comorbid conditions and treated as per the recommended guidelines. About two-thirds of the participants were aged 50 and above, of which more than half were illiterates, and 93.5% were living below the poverty line. Majority of ulcers were seen on the forefoot; with the head of meta-tarsal bone 27 (41.6%) as the commonest site, followed by calcaneum 23 (38.3%) and great toe 10 (16.6%). Mean ulcer depth was 0.61 (0.57) cm, the area was 5.24 (6.73) cm^2^ and ulcer volume was 4.72 (14.33) cm^3^. Ulcer dimensions were significantly associated with low body mass index, hypertension and smoking.

**Conclusions/Significance:**

Identifying the risk factors delaying wound healing and detailed assessment of ulcers are of profound importance to predict the outcome of plantar ulcers in leprosy patients. The study findings indicate the need for better policies by the leprosy control program for the comprehensive management of plantar ulcers.

## Author summary

Plantar ulceration is a major cause of disability in leprosy patients. Most of the ulcers are chronic, which occur even years after completion of Multidrug therapy (MDT) and do not heal despite proper wound care and dressings. Understanding the factors associated with non-healing of plantar ulcers and identifying appropriate control measures could be one of the major interventions for preventing disability in leprosy patients. We undertook a study on 66 leprosy patients with plantar ulcers in these lines. Most of the ulcers were seen on the forefoot, with meta-tarsal bone involvement, the most vulnerable pressure point, indicating the necessity for its protection to prevent the occurrence of ulcers. Key findings from this study are a significant association of increased ulcer dimensions with low body mass index, hypertension and smoking. Further studies are required to establish the role of these comorbidities in causation and progression of ulcer.

## Introduction

Leprosy, a chronic infectious disease caused by the *Mycobacterium leprae*, continues to be one of the public health challenges for countries like India, which accounts for more than half of the world’s new leprosy cases [1]. Dysfunction of peripheral nerves resulting in skin anaesthesia is one of the salient clinical manifestations of leprosy, which further leads to disabilities, including ulcers [2]. Plantar ulcers are one of the commonest complications of leprosy leading to Grade 2disability (G2D) and occur in about 10% to 20% of patients [3]. Recently launched global leprosy strategy of WHO aims to reduce the number of G2D among new cases. The current G2D rate stands at 1.7 per million populations, with India accounting for 39.8% of cases [4]. Plantar ulcers are typically painless and increase in size without healing for weeks to months. On treatment, an acute ulcer may heal but, sometimes, the ulcer can recur and persist as a small raw area called chronic ulcer [5]. Chronic ulcers are characterised by hyperkeratotic edges with fibrosed base and floor covered with pale granulation tissue with scanty discharge [2]. Long-standing and untreated plantar ulcers can undergo malignant changes [6].

Plantar ulcers in leprosy are in part caused by peripheral neuropathy and/or a single vascular disturbance [7,8]. Hence any other underlying comorbidity in leprosy patients which can cause neuropathy or vascular disturbance, such as diabetes or hypertension would pose a higher risk for development of chronic leg ulcers and may have implications on the healing of ulcers. It may hence also be important to address such comorbidities for comprehensive ulcer care in leprosy patients. We, therefore, aimed to study the association of comorbid conditions such as malnutrition, hypertension, and diabetes as possible risk factors for severity and non-healing of plantar ulcers in leprosy patients. Knowledge accrued from the study is expected to provide new leads for better care for plantar ulcers in leprosy.

## Methods

This is a cross-sectional study conducted at LEPRA Society-Blue Peter Public Health and Research Center (BPHRC), Hyderabad, India from June 2018 to June 2019. Leprosy patients of age 20–65 years of both sexes presented with plantar ulcers were included after obtaining informed consent. All the studied patients had plantar ulcers at the time of the study. A pretested questionnaire was administered to the participants in the study. Detailed clinical examination was done according to the National Leprosy Eradication Program (NLEP) guidelines with reference to ulcer and other leprosy related consequences. Characteristics of the ulcers, including severity, size, discharge, were recorded. Patients not willing to participate were excluded. Ulcers were graded as per the National Pressure Ulcer Advisory Panel (NPUAP) guidelines. Patients were screened for underlying comorbid conditions–hypertension (through measurement of BP), diabetes (fasting plasma sugar or random blood sugar testing), Anemia (haemoglobin), nutritional assessment (BMI and CED), smoking and alcohol consumption. All the patients were managed as per the standard guidelines (NLEP); besides, all the infected ulcers were treated with antibiotics, guided by culture and antimicrobial susceptibility tests.

### National Pressure Ulcer Advisory Panel (NPUAP) staging [9]

Ulcers were graded according to NPUAP staging to define the evolutionary stages of the ulcers, denoted in grades: I (intact skin with non-blanching erythema), II (partial-thickness loss of skin with exposure of the dermis), III (full-thickness loss of skin) and IV (full-thickness loss of skin and loss of tissue).

### Ulcer measurements

Ulcer size was measured in terms of length, breadth, and depth with cotton-tipped applicators and disposable scales. Wound area was calculated using the formula for an ellipse: Length × width × 0.7854 (an ellipse is closer to a wound shape than a square or rectangle that would be described by simple length × width) and the volume was calculated using the formula (length × width × 0.7854) × depth as per the recent literature published on wound measurements[10].

### Definitions

Non-healing ulcer [11]: Ulcer failure to show signs of healing of at least 4 weeks of treatment with complete rest and sterile dressings.

Hypertension: defined as systolic BP of at least 140 mm Hg or diastolic BP of at least 90 mm Hg

Diabetes: defined as having a high fasting plasma glucose reading (≥126mg/dL [7.0mmol/L] or ≥200 mg/dL [11.1 mmol/L] if patients reported not fasting

Anaemia: defined as haemoglobin (Hb) <13 g % in males and 12 g % in females

Body mass index (BMI): calculated using the following formula: weight (kg)/height (m^2^).

#### Chronic energy deficiency (CED) [12,13]

Chronic energy deficiency (CED) was calculated based on BMI<18.5kg/m^2^.

All the clinical parameters of the ulcers were studied vis a vis the comorbidities.

#### Statistical analysis

R programming software (Version 3.0.1) was used for data analysis. Mean, and the standard deviation was calculated for continuous variables. Frequencies were calculated for categorical variables. Kruskalwallis test to assess differences between the groups for non-normal continuous variables. A generalized linear regression model was carried out to with risk factors as the independent variable and wound volume as a dependent variable with and without adjustment for age and sex. p-value less than 0.05 was considered significant.

### Ethics statement

The study protocol was approved by the institutional ethics committee of LEPRA Society-Blue Peter Public Health and Research Center (BPHRC), Hyderabad, India. All the participants were adults and included after obtaining written informed consent.

## Results

### Socio-demographic

A total of 66 leprosy patients (male/female = 51/15) with plantar ulcer who were recruited into the study were analysed. Table 1 shows the socio-demographics of the participants. About two-thirds of the participants were aged 50 and above, more than half of them were illiterates, and 93.5% were living below the poverty line (BPL card present). Deformed foot, decreased the size of the foot (absorption) with excessive walking seems to be the most common cause of the breakdown of plantar ulcer as evidenced with 37.9% of participants were manual labourers. Eight (12.1%) patients had difficulties in working, due to plantar ulcers of which, five (62.5%) patients had to stop working due to the presence of an ulcer. Fifty-three patients (80.3%) reported out of pocket expenditure in terms of travel and medication before reporting to our centre, indicating the cost as the barrier to access to services.

**Table 1:** Distribution of patients with sociodemographic characteristics.

Variables	n = 66
Age in years (mean (SD))	52.71 (13.15)
Sex Males (%) Females (%)	51 (77.3) 15(22.7)
Marital Status (%)	
Married	60 (90.9)
Separated	1 (1.5)
Un Married	2 (3.0)
Widow	3 (4.5)
Religion (%)	
Christian	8 (12.1)
Hindu	55 (83.3)
Muslim	3 (4.5)
Literacy state of the patient (%)	
Degree	1 (1.5)
Graduation and above	1 (1.5)
High School	12 (18.2)
Illiterate	41 (62.1)
Intermediate	4 (6.1)
Primary/Upper Primary	7 (10.6)
Occupation of the patient (%)	
Business/ Self Employed	11 (16.7)
Housewife	2 (3.0)
Laborer	25 (37.9)
Other	22 (33.3)
Professional	1 (1.5)
Unskilled worker	5 (7.6)
Age	
less than 30 years	3 (4.5)
30–39 years	8 (12.1)
40–49 years	11 (16.7)
50–59 years	21 (31.8)
More than 60 years	23 (34.8)

### Status of MDT treatment and other leprosy related sequelae

Sixty-one patients (92.4%) completed Multidrug therapy (MDT), five (7.5%) patients were on MB MDT at the time of enrolment. Two (3%) patients out of 66 were in Type 2 lepra reaction.

### Comorbidities and other risk factors

Hypertension and diabetes were seen in 12.3% and 10.8%patients, respectively. The mean BMI was 22.8kg/m^2^. Anaemia was seen in 39.3%while, leukocytosis was noted in 27.2% of patients. Less than 10% were consuming alcohol, while about a third were using tobacco.

### Ulcer characteristics (Table 2)

**Fig 1:**
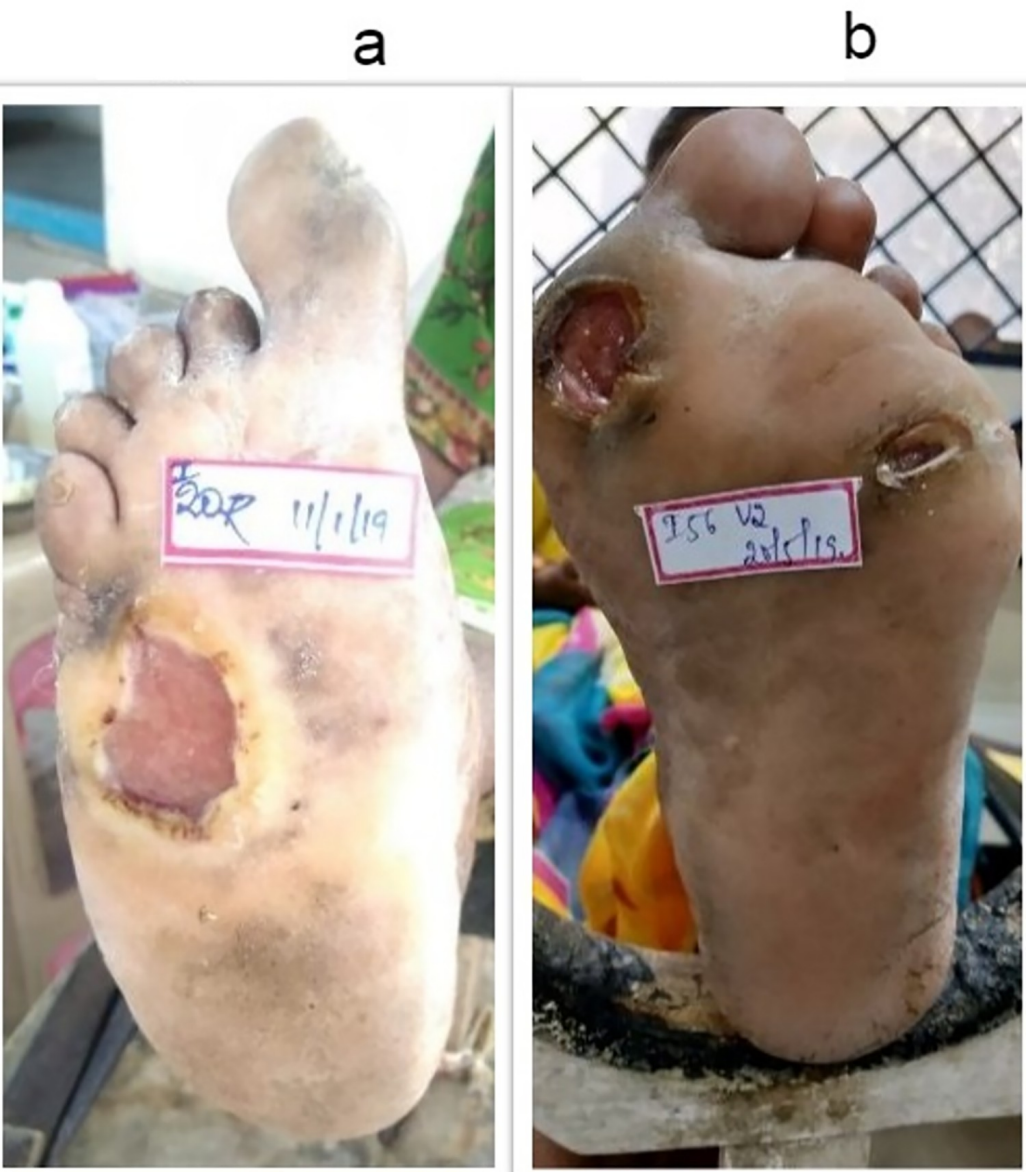
Image showing single (Fig 1a) and multiple ulcers (Fig 1b) belonging to NPUAP stage III.

Sixty-three patients had a single ulcer and three patients with two concomitant ulcers making it a total of 69 ulcers. The duration of ulcer ranged from one month to 240 months with mean duration 1238.82 (2159.39) days. In 25 (37.9%) patients, blisters were present on the pressure points before the development of ulcers. Majority of ulcers were seen on the forefoot; with the head of metatarsal bone 27 (41.6%) as the most frequent site, followed by calcaneum 23 (38.3%) and great toe 10 (16.6%). Mean ulcer depth was 0.61 (0.57) cm, the area was 5.24 (6.73) cm^2^ and ulcer volume was 4.72 (14.33) cm^3^. The majority (79.4%) of ulcers belonged to NPUAP stage III (Fig 1).

**Table 2:** Characteristic of Ulcer.

Variables	n = 66
Duration of ulcer (in years) (%)	
< 1	29 (46.8)
1–5	22 (35.5)
5–10	8 (12.9)
>10	3 (4.8)
Depth of ulcer (mean (SD))	0.61 (0.57)
Wound area (mean (SD))	5.24 (6.73)
Wound volume (mean (SD))	4.72 (14.33)
Ulcer site (%)	
Greater toe	9 (14.1)
Heel	23 (35.9)
Metatarsal	25 (39.1)
Mid foot	7 (10.9)
NPUAP staging (%)	
II	10 (15.9)
III	47 (79.4)
IV	3 (4.8)

The serous discharge was observed in 60 (90.9%) and purulent discharge in seven (10.6%) ulcers while, bloody and serosanguinous in one (1.5%) ulcer each. The draining lymph nodes were tender and enlarged in two (3%) patients. Cauliflower like growth arising from the ulcer suggestive of Squamous cell carcinoma (SCC) was observed in two (3%) patients. Asymptomatic, deep, non-healing fissures on heels and toes observed in 28 (42.4%) patients. Callosities noted in 31 (47.0%) patients.

Fifty (72.4%) ulcers were positive for pathogenic bacteria on swab cultures were treated with a full course of appropriate antibiotic basing on the anti-microbial susceptibility tests and followed up.

### Ulcer dimensions and risk factor association

**Fig 2:**
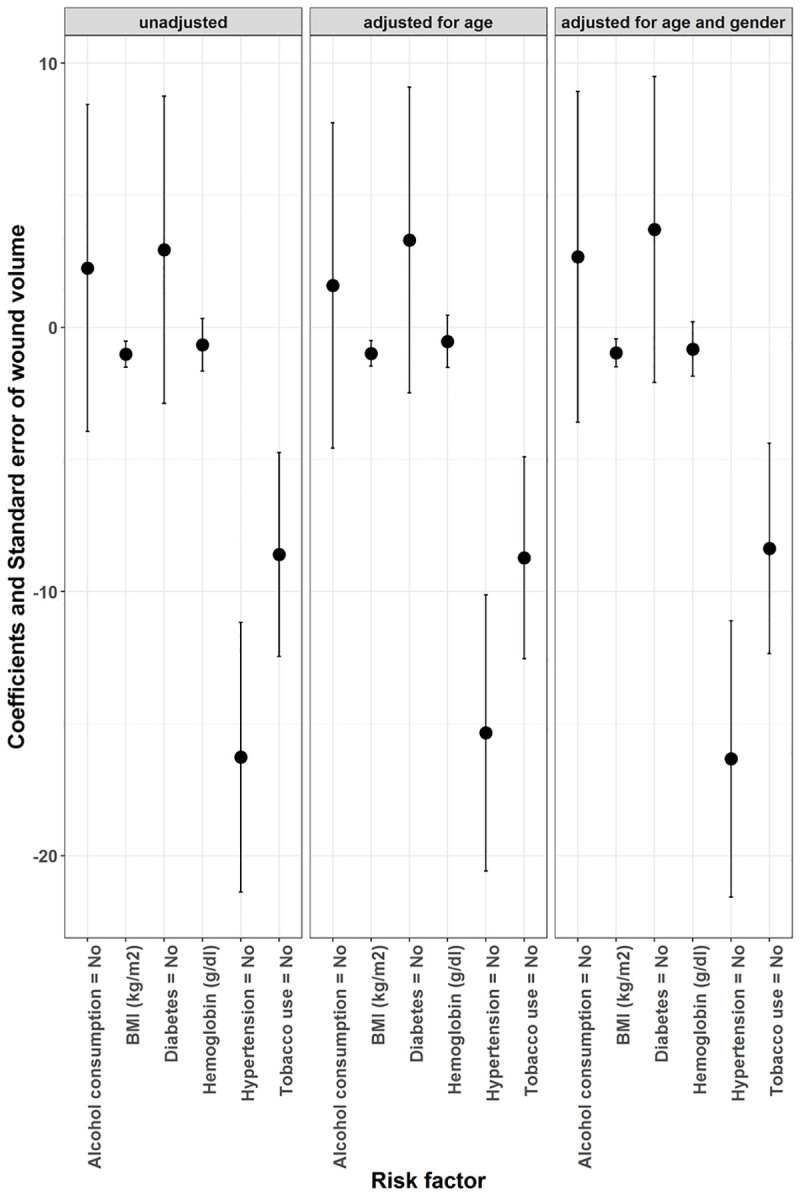
A generalized linear regression model with wound volume as a dependent variable and risk factors as an independent variable in adjusted and unadjusted models.

When examining the correlation between BMI, comorbidities, smoking and alcoholism with the leprosy plantar ulcers, we found that greater depth and volume was significantly associated with CED (BMI <18.5), hypertension and history of smoking. Although hypertension and smoking seem to be significant predictors of wound volume in both adjusted and unadjusted models (Fig 2). There was a considerable difference in ulcer depth, area and volume between ulcers found on the heel in comparison to those on other anatomical regions. However, the association is not statistically significant. There were no significant differences observed in wound dimensions in patients with and without positivity of wound swab culture for pathogenic bacteria.

## Discussion

EW Price introduced the term ‘plantar', ulcer and defined it as "chronic ulceration of the anaesthetic sole of the foot, situated in well-defined areas overlying bony prominences, resistant to local or systemic therapy and characterised by a marked tendency to recurrence” [14]. The moment ulceration occurs, this foot becomes ‘ulcer prone', and the vicious cycle of scar-ulcer-scar takes place [2].

Reported in this study, most patients with plantar ulcers were a male, which is in concordance with the results of Oliveira et al. [7], Gaschignard et al. [15] and Barreto and Salgado [8]. Majority of patients in this study is above 50 years (66.6%) and affected patients ‘work capacity and social life (Table 1). 12.1% of patients with plantar ulcers had difficulties at work, as observed by Femina et al. [16].

We found that the majority of patients studied had normal BMI, in line with the report from Wagenaar It et al. [17]. However, patients with BMI < 18.5 had demonstrated increased ulcer dimensions(Table 3). In a report by Oliveira et al., leprosy patients with and without plantar ulcers, were found to be either overweight or obese [7]. These findings prompted us to infer the changing trend in the nutritional status among leprosy patients.

**Table 3:** Risk factor analysis with wound dimensions.

	n	Depth of wound	Wound area	Wound volume
**BMI categories**				
Less than 18.5	5	1.60 (1.47)	8.13 (11.41)	26.21 (47.47)
18.5 to 24.9	53	0.54 (0.34)	5.09 (6.08)	3.02 (5.10)
25 to 29.9	0			
30 and above	2	0.75 (0.35)	2.45 (3.19)	1.28 (1.53)
**p-value**		<0.001	0.514	0.003
**Comorbidities**				
Diabetes	7	0.43 (0.28)	3.32 (4.14)	2.17 (4.26)
Hypertension	8	1.10 (1.29)	8.23 (9.56)	18.99 (38.24)
No diabetes and hypertension	51	0.56 (0.35)	5.12 (6.51)	2.80 (3.91)
**p-value**		0.029	0.348	0.01
**Other risk factors**				
Alcoholism	7	0.42 (0.34)	5.86 (6.56)	2.68 (3.73)
Smoking	18	0.92 (0.97)	6.91 (8.68)	10.89 (26.03)
No alcohol consumption and no smoking	41	0.50 (0.17)	4.40 (5.72)	2.24 (3.17)
**p-value**		0.021	0.417	0.096
**Ulcer site**	64	0.61 (0.57)	5.24 (6.73)	4.72 (14.33)
Greater toe	9	0.40 (0.12)	2.88 (4.95)	1.25 (2.50)
Heel	23	0.78 (0.84)	5.54 (7.23)	7.94 (23.28)
Meta-tarsal	25	0.57 (0.39)	5.18 (7.10)	3.24 (6.41)
Midfoot	7	0.46 (0.11)	6.61 (6.37)	3.30 (3.19)
**p-value**		0.283	0.709	0.594
**Anaemia**				
Yes	26	0.63 (0.70)	5.63 (7.52)	6.70 (21.35)
No	36	0.59 (0.45)	5.00 (6.56)	3.44 (6.40)
**p-value**		0.826	0.733	0.402
**Positivity of wound swab**				
**Yes**	47	0.62 (0.61)	4.69 (6.00)	4.86 (16.45)
**No**	19	0.61 (0.45)	6.53 (8.25)	4.37 (7.53)
**p-value**		0.936	0.321	0.902

Fifty (72.4%) out 69 ulcers investigated in 47 patients were positive for one of the pathogenic bacteria; Staphylococcus aureus, Staphylococcus haemolyticus and Proteus mirabilis has been found to be most frequent pathogens; were treated with a full course of appropriate antibiotic basing on the anti-microbial susceptibility tests and followed up. Ampicillin, cefepime, amoxiclav have been found to be most sensitive antibiotics.

Wound healing requires oxygenation. Due to anaemia, the oxygen-carrying capacity of blood is halted, leading to impaired healing [18]. We found that 39.3% of patients studied were anaemic, indicating the importance of considering anaemia as one of the risk factors for non-healing ulcers (Table 3). Further studies are needed to understand the role of anaemia in recurrence of ulcers and the role of anaemia correction in preventing ulcer recurrence.

As it is expected, most of the ulcers were located on metatarsal and calcaneal regions of foot that bears weight during walking and standing [19]. Loss of arch of the foot can predispose to midfoot ulcers as evidenced in 10% of study participants. Three-fourths of the study participants were found to be having NPUAP stage III, indicating the risk of non-healing and chronicity [20].

In the present study majority (84.0%) of ulcers were chronic wounds which resulted from delayed healing and recurrence. Plantar anaesthesia, barefooted walking, poor quality of scar formation resulting from previous ulceration, excessive load on the scar, and secondary infections are already known as causative factors for ulcer recurrence and have also been observed in the study participants [21].

Measurements of ulcer dimensions as followed in our study, helped in well characterizing the ulcers for clinical management and assessing the therapeutic outcome. Hence we emphasise the usefulness of routine measurements of ulcer dimensions as a simple clinical tool which should be followed at all levels of ulcer care settings.

It is well known that cigarette smoking is a key risk factor that halts the healing of any ulcer [2]. This is the first time that such finding has been made on leprosy plantar ulcers. Nicotine, a vasoconstrictor in the smoke reduces nutritional blood flow to the skin, leading to tissue ischemia and impaired healing [22]. Cigarette and bidi (hand-rolled cigarettes in India) smoking were seen in 18.2% and 9.1% of the study participants. We found that smokers had significantly greater ulcer dimensions than alcoholics and those who do not smoke and consume alcohol(Table 3).

We found that patients with hypertension had greater ulcer dimensions indicating the need for active screening and management of hypertension in leprosy patients, to prevent progression of plantar ulcers (Table 3). Studying the association of treatment of hypertension with wound healing is not under the scope of the current report. We have adjusted for age and gender as the other covariates might have been influenced by age (diabetes and hypertension increase with age) and gender (Tobacco and alcohol use is more common in males than females).

Both diabetes and leprosy are known to cause neuropathic ulcers on foot [23]. About 10% of leprosy patients studied were also diabetic (Table 3). Screening for diabetes in leprosy patients is usually not done since leprosy is considered as a disease of under-nutrition and low socio-economic status, while Type II diabetes is considered as a lifestyle disease associated with over nutrition. India, where both diabetes and leprosy are common, clinicians should consider them in their differential diagnosis, especially when presented with complications such as neuropathic ulcers [24].

**Fig 3:**
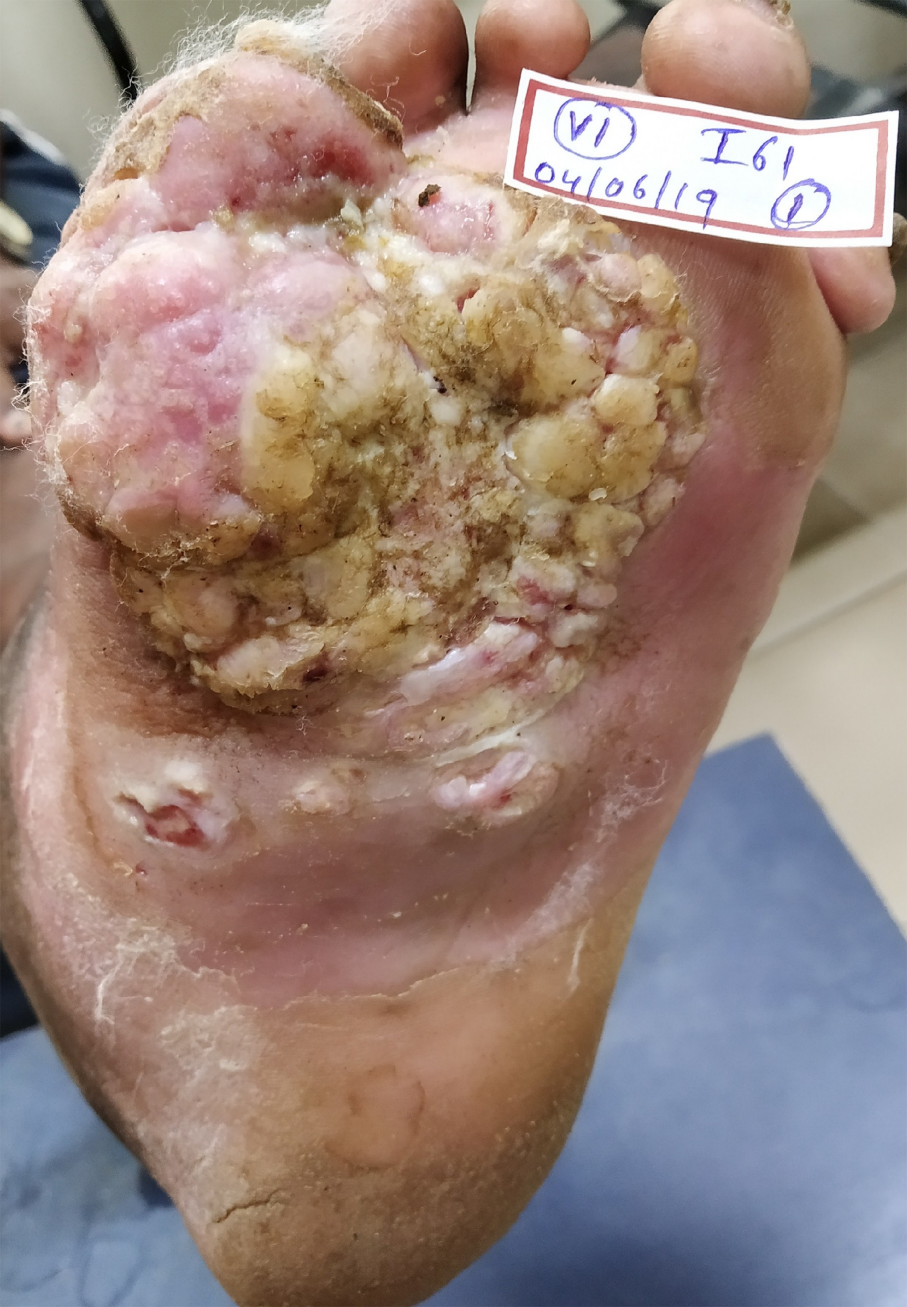
Image showing cauliflower growth arising from chronic plantar ulcer.

Another interesting finding from our study is the observation of cauliflower-like growth on ulcers suggestive of squamous cell carcinoma (SCC) in 3% of the patients studied (Fig 3). Repeated trauma secondary to sensory loss and chronic inflammation is known to contribute to the malignant transformation of long-standing ulcers. The incidence rate of development of SCC in patients with leprosy is reported as 0.79:1000.4 annually [25]. The development of suggestive SCC, even in a small proportion of patients emphasizes the need for better interventions for preventing ulcers or at least preventing the recurrence and chronicity of existing ulcers, under the standard DPMR policies of the leprosy control program.

The study is conducted on a small sample size. Some data points were missing for a few participants who were leprosy treated a long time ago. The current study was a cross-section design which could only associate the comorbidities with ulcer healing as a preliminary finding. The study findings would have further improvised had there been a case-control study with larger sample size.

Often leading to complications, plantar ulcers remain a significant cause of economic and social impact of leprosy and occur even after patients have been completely treated with MDT. Identifying the factors that delay or enable the healing of ulcers is of profound importance for clinical management. Observations from the current study necessitate detailed clinical assessment and follow-up of ulcers. The study findings also indicate the importance of screening for underlying medical conditions such as hypertension, diabetes, anaemia, and undernutrition, which have negative implications on wound healing. The multipronged approach through medical intervention for ulcer care, preventive screening for associated risk factors and patient counselling for healthy lifestyle help in reducing the morbidity associated with leprosy plantar ulcers. This, in turn, positively impacts the quality of life of leprosy-affected people and their social and economic wellbeing.

## Supporting information

S1 Checklist Leprosy ulcer STROBE checklist. (DOC)
